# Nu-DESC DK: the Danish version of the nursing delirium screening scale (nu-DESC)

**DOI:** 10.1186/s12912-017-0271-x

**Published:** 2017-12-29

**Authors:** Daniel Hägi-Pedersen, Kasper Højgaard Thybo, Trine Hedegaard Holgersen, Joen Juel Jensen, Jean-David Gaudreau, Finn Michael Radtke

**Affiliations:** 1 Department of Anaesthesiology, Næstved Hospital, Ringstedgade 61, 4700 Næstved, Denmark; 2 Hospital Management, Næstved Hospital, Næstved, Denmark; 3 Language- and integration centre, Næstved, Denmark; 4 Faculty of Pharmacy, Laval University, QC, Québec Canada; 5 Department of Anaesthesiology and Surgical intensive care medicine, Campus-Virchow-Klinikum and Campus Charité Mitte, Charité - Universitätsmedizin Berlin, Berlin, Germany

**Keywords:** Nursing delirium screening scale, Confusion, Delirium, Screening

## Abstract

**Background:**

Delirium is one of the most common complications among elderly hospitalized patients, postoperative patients and patients on intensive care units with a prevalence between 11 and 80%. Delirium is associated with higher morbidity and mortality. Reliable instruments are required to detect delirium at an early time point. The Nursing-Delirium Screening Scale (Nu-DESC) is a screening tool with high sensitivity and good specificity. However, there is currently no official translation after ISPOR guidelines of any Danish delirium assessment tools available. Thereby hampering the implementation of 2017 ESA-Guidelines on postoperative Delirium in the clinical routine. The aim of this study is to provide an official translation and evaluation of the Nu-DESC into Danish following the ISPOR process.

**Methods:**

The Nu-DESC was translated after International Society for Pharmacoecomonics and Outcome Research (ISPOR) guidelines to Danish after permission of the original author, and is evaluated by medical staff and finally approved by the original author.

**Results:**

All steps of the ISPOR guideline were consecutively followed, without any major problems. The evaluation of the Nu-DESC DK regarding its intelligibility and feasibility showed no statistically significant differences between nurses and medical doctors ratings. The translation was authorized and approved by the original author.

**Conclusion:**

This study provides the Nu-DESC DK, an official Danish delirium screening instrument, which can detect all psychomotor types of delirium.

## Background

Delirium is one of the most common complications among elderly hospitalized patients. The reported prevalence is between 11% to 50% in medical and surgical populations, and up to 80% patients receiving intensive care [1–3]. Delirium is associated with a high morbidity and a threefold 6-month mortality [4, 5]. Patients with delirium have higher rates of complications as well as a higher necessity for rehabilitation. It is reported that patients with delirium during hospital stay often have a lower cognitive function one year after discharge [6, 7].

Studies have shown that up to 84% of all delirium cases are not detected in daily clinical routine [8, 9]. Furthermore, delirium is reported to be preventable in up to 40% of patients [10, 11] The lack of availability of a fast, adequate and evaluated Danish delirium screening instrument divests clinicians of using delirium, as a quality improvement measure as it is recommended in guidelines [11, 12].

There are three subtypes of delirium described.

-The **hyperactive delirium** is characterized by psychomotor hyperactivity and an increased response to stimulation (1.6%) [13].

-The **hypoactive delirium** is characterized by psychomotor retardation and a reduced response to stimulation (43.5%). [13].

-The **mixed type** has symptoms from both the hypoactive and the hyperactive form and is the most common subtype (55%) [14].

Especially, the hypoactive form of delirium has been described as being associated with more negative outcomes [15]. Yet, without an adequate delirium instrument foremost patients with hypoactive delirium are often not diagnosed [16].

Gaudreau et al. have developed the nursing delirium screening scale (Nu-DESC) as a nurse based diagnostic instrument for delirium with special focus on hypoactive delirium. In a validation study of 146 patients the Nu-DESC showed a high validity (sensitivity 86%, specificity 87%). The average time used for screening per patient was less than a minute [17, 18], thereby ensuring high feasibility in clinical routine. In relation to the DSM-IV criteria, which were the gold standard for diagnosing delirium, the Nu-DESC has shown good validity [17]. Furthermore it was suggested that the Nu-DESC not only recognizes significantly more cases of delirium, but detects them at an earlier timepoint [19] Additionally, the Nu-DESC can detect hypoactive delirium due to the fifth element focusing and evaluating the psychomotor activity of patients.

The nursing staff spends more time with the patients than other medical professionals. Therefore choosing a nurse based screening instrument is an obvious and important part of implementing Delirium management into the clinical routine [20].

Presently, there is no Danish delirium screening scale available, that has been officially translated to Danish according to the standard of ISPOR guidelines.

### Aim and objectives

The aim of this study was to establish an ISPOR guidelines conform translation and evaluation of the Nu-DESC in Danish. Adhering to the recommendations of the Translation and Cultural Adaptation Group of Patient Reported Outcomes (PRO) measures – Principles of Good Practice (PGP) [21] thereby providing a reliable and easy to use delirium screening scale for use in research and clinical routine in Denmark.

## Methods

We choose to translate Nu-DESC after published guidelines [21]. The evaluation of the instrument will be performed by clinicians of two different departments.

### Setting and procedure

The translation and debriefing procedure was performed at the Department of Anaesthesiology, Næstved Hospital, Denmark. The evaluation took place at the Department of Anaesthesiology and Department of Orthopaedic Surgery, Næstved Hospital, Denmark. We followed the International Society for Pharmacoecomonics and Outcome Research (ISPOR) guidelines [21] in order to have a reproducible translational process. The translation procedure was defined in advance, with all the steps of the translation and evaluation process. The team of translators defined the timeframe and administrative aspects as well as performed the translation and the evaluation of the instrument.

### The translation process

The International Society for Pharmacoecomonics and Outcome Research (ISPOR) has set up a task force for the development of guidelines for translation and cultural adaptation of patient-reported outcome measures [21]. After reviewing 12 official guidelines, including the World Health Organization (WHO) standard, they published a report for scientific accurate practice for translation of measuring instruments. The 10 steps of the ISPOR guidelines are shown in Table 1.

**Table 1:** Steps of ISPOR guidelines for translation

Steps		
1	Preparation	Obtain permission to use instrument and involvement of original author
2	Forward Translation	Development of at least two independent forward translations
3	Reconciliation	Reconciliation of the forward translations into a single forward translation
4	Back Translation	Back translation of the reconciled translation into the source language
5	Back Translation Review	Review of the back translations against the source language
6	Harmonization	Harmonization of all new translations with each other and the source version
7	Cognitive Debriefing	Cognitive debriefing of the new translation, with 5–8 healthcare professionals
8	Review of Cognitive Debriefing Results and Finalization	Cognitive debriefing results are reviewed and the translation finalize
9	Proofreading	The finalized translation is proofread
10	Final Report	Report is written on the development of the translation

### Statistics

The following characteristics of the participants were documented: gender, occupational group, clinical working place. Mann-Whitney-U-Test was performed for differences between the professional groups. Data was analysed with SPSS version 21 (SPSS, Chicago, IL, USA).

## Results

All ISPOR recommended steps were followed consecutively. And no relevant problems were encountered at any step.

### Preparation

After agreement and consent from Jean-David Gaudreau the translation process into the Danish language was started.

### Forward translation

After initial distribution of the tasks, three medical doctors and one nurse performed three separate forward translations from English to Danish, all independently and without interference from one another.

### Reconciliation

The translations were then merged in one preliminary version. The Danish version was adapted to a clinical setting without changing the meaning. Each phrase was discussed carefully, compared between the four translations to find the most accurate and fitting phrase. The final wording was consented, when all translators after agreed unanimously. The phrase “reaction is deferred” was one of the phrases, which challenged the translators to find the clinically appropriate phrase in Danish. Several different wordings were tested in the process finally honing in on one clinically phrase that all translators consented to unanimously.

### Back translation

The preliminary version was then back translated into English by an experienced and certified language teacher without knowledge to the original English version. This back translation was then sent back to the authors.

### Back translation review

The back translation of the preliminary Danish version was then thoroughly compared with the original text in regard to the necessity of performing adjustments. This back translation showed no substantial deviations from the original after close comparison and assessment performed by the translating authors.

### Harmonization

The English back translation was then sent to the original author Jean-David Gaudreau for inspection and final approval. Jean-David Gaudreau assessed the back translation in respect to conformity of content and language as well as in regard to the agreement with the original version. The final step of the translation was then approved.

### Cognitive debriefing

The authorized Danish version underwent a structured evaluation process by clinical staff. The evaluation team consisted of 20 nurses and doctors from two different specialties and wards (Table 2). All medical doctors were employees of the department of anaesthesiology (3 consultants and 1 resident). The nurses were all registered nurses, with several years of clinical experience. All staff evaluating the Nu-DESC got the Nu-DESC DK on paper and an evaluation form to rate the instrument.

**Table 2:** Characteristics of the participating staff of the cognitive debriefing

		Frequence	Percent
Sex	Male	3	15.0
	Female	17	85.0
Professional group	MD	4	20.0
	Nurse	16	80.0
Working place	Orthopaedic ward	10	50.0
	Recovery room	10	50.0

### Review of cognitive debriefing results and finalization

The individual items of the test were evaluated in regard to understand ability, both in language and content. In addition, feasibility and usability were evaluated. Each item was rated on a 6 step Likert scale (strongly agree, agree, slightly agree, slightly disagree, disagree, and strongly disagree). The evaluation of the Nu-DESC DK performed by the two professional groups and two different specialties showed no fundamental difficulties in relation to content or language. The evaluation also revealed that all elements are readily usable.

There were no significant differences between the evaluations of the nursing staff and the medical doctors (Table 3).

**Table 3:** Results of the cognitive debriefing Mann-Whitney U test

Item		Criteria	Professional Group		*P*-Value
			MD	Nurse	
Disorientation	Understanding	Language	4,75	3,94	0,290
		Content	5,25	4,19	0,099
	Feasibility	Time	5,50	5,00	0,641
		Usability	5,33	4,56	0,487
Inappropriate behaviour	Understanding	Language	4,75	5,31	0,820
		Content	4,75	5,31	0,820
	Feasibility	Time	5,00	5,31	0,437
		Usability	5,25	5,40	0,810
Inappropriate communication	Understanding	Language	4,50	4,94	0,494
		Content	5,00	4,94	0,892
	Feasibility	Time	4,75	5,27	0,411
		Usability	4,75	5,00	0,810
Illusions/Hallucinations	Understanding	Language	5,75	5,50	0,963
		Content	5,75	5,50	1000
	Feasibility	Time	5,75	5,56	0,682
		Usability	5,75	5,60	0,810
Psychomotor retardation	Understanding	Language	5,75	5,06	0,494
		Content	5,75	5,19	0,494
	Feasibility	Time	5,75	5,19	0,335
		Usability	5,75	5,07	0,357

Assessment of the test items relative to understanding and feasibility. Rating on a 6 step scale (strongly agree, agree, slight agree, slight disagree, disagree, strongly disagree). Presented are the means of the evaluation of the two groups. Analysis of the group difference of the single items with Mann-Whitney-U-Test. Statistic significance level *p* < 0,05

### Proofreading

The finalized version was proofread by three of the translators.

### Final report

Three of the authors reviewed the result as well as the whole process of translation. After the final positive evaluation and due to the good results of the cognitive debriefing the authors approved the Danish version of the Nu-DESC thereby completing the process suggested by the ISPOR guidelines.

**Fig. 1:**
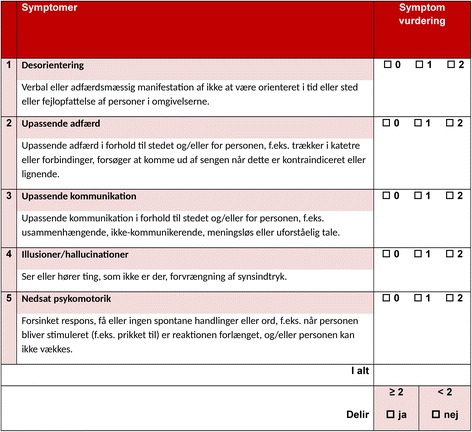
Nu-DESC DK (Nursing-DElirium SCore Denmark)

The final and approved version of Nu-DESC DK (Fig. 1) is now ready for clinical and scientific use in Danish speaking countries.

## Discussion

This study provides the first official Danish translation of a delirium screening instrument according to the ISPOR guidelines. We followed the guideline recommendations thoroughly. A consecutive evaluation of the Nu-DESC DK by potential future users showed most promising results in regard to clearness of wording and feasibility.

Our process of the translation procedure followed the guidelines of the Translation and Cultural Adaption Group (TCA Group) and the authors had contact with the author of the original instrument. The process of the ISPOR guidelines guarantees that the translation preserves the concept and the intention of the original instrument.

With permission of the original author, the translation after the Guidelines of the Translation and Cultural Adaptation Group (TCA) and after the principles of the International Society for Pharmacoeconomics and Outcome Research (ISPOR) [21] was done to Danish.

Our evaluation showed that the Nu-DESC DK is comprehensible and feasible. There was no significant difference between the evaluation results from the nursing staff and medical doctors’, which suggests that the tool is equally usable by both groups.

The Nu-DESC DK is, to the best of our knowledge, the first Danish delirium screening tool translated from the original language following ISPOR-guidelines. It has been authorized by the original author and is free for all interested parties to use.

Delirium is the most common psychiatric diagnosis in elderly hospitalized patients as well as in the postoperative setting and on intensive care units, with prevalence’s from 11% to 50% [1–3]. The diagnosis of delirium can be challenging due to the different forms of delirium, and due to the fact that delirium can fluctuate during the course of a day. That is the reason why in the daily clinical routine the detection rate of delirium is usually low [8, 9]. The nursing staff plays a central role in detecting delirium [20]. The availability of an easy to use, nurse based delirium instrument is the prerequisite for a widespread implementation.

In Denmark and specifically in our department there is a need for an easy to use Danish version of the Nu-DESC, especially since it has been recommended in the 2017 guidelines of the European Society of Anaesthesiologists on postoperative delirium [12].

Often the quality of data gathered through translated measurement instruments is dependent of the translation process [21]. It is worth noting the inconsistency and lack of methodology of the majority of translations in this field.

We therefore support the ISPOR principles [21]. Only systematic procedures and subsequent evaluations can produce valid and reliable measurement instruments.

As a limitation to our study the lack of a DSM 5 validation could be stated. However, we did not choose to compare the Nu-DESC DK to DSM IV or DSM 5 criteria because an isolated comparison between the Nu-DESC DK and the “new” DSM 5 criteria did not seem meaningful (other Nu-DESC translations and Delirium tools where only compared to DSM IV) and due to the fact that the whole delirium focus has shifted significantly with the progression of delirium gold standards from DSM IV to DSM 5. Now there is only a 30% overlap between DSM IV and 5 in delirium diagnosis using the strict definition [22, 23]. Additionally, the Nu-DESC has been abundantly validated in comparison to DSM IV in numerous different languages and settings [24–28].

An international multicentre validation study according to DSM 5 criteria (strict as well loose interpretation of DSM 5 criteria) would be very helpful. However it was not the primary aim this ISPOR conform translation.

## Conclusion

With the Nu-DESC DK we provide an official Danish delirium screening instrument, which can detect all psychomotor types of delirium. Nu-DESC DK has the potential to be the cornerstone to the screening and diagnosis of delirium in Denmark.

## Abbreviations

ISPOR: International Society for Pharmacoecomonics and Outcome Research
Nu-DESC DK: Nursing Delirium Screening Scale Denmark
Nu-DESC: Nursing Delirium Screening Scale
TCA Group: Translation and Cultural Adaption Group
WHO: World Health Organization
